# Spatial pattern completion deficits in older adults

**DOI:** 10.3389/fnagi.2013.00003

**Published:** 2013-02-13

**Authors:** Meera Paleja, Julia Spaniol

**Affiliations:** ^1^Department of Neuropsychology and Neurosurgery, Montreal Neurological Institute, 3801 Rue UniversityMontreal, QC, Canada; ^2^Department of Psychology, Ryerson UniversityToronto, ON, Canada

**Keywords:** spatial pattern completion, navigation, aging, episodic memory, hippocampus

## Abstract

Aging may have an impact on the CA3 autoassociative network of the hippocampus, posited by computational models as supporting pattern completion. Twenty-five young (YAs) and 25 older adults (OAs) performed a spatial pattern completion task using a computerized navigational paradigm analogous to a rodent pattern completion task reliant on the CA3. Participants identified a previously seen goal location, and the availability of distal cues in the environment was manipulated such that 0, 2, or 4 cues were missing. Performance in both groups declined as a function of decreased cue availability. However, controlling for age differences in task performance during a pre-experimental baseline task, OAs performed equivalently to YAs when all cues were available, but worse than YAs as the number of cues decreased. These findings suggest spatial pattern completion may be impaired in OAs. We discuss these findings in the context of a growing body of literature suggesting age-related imbalances in pattern separation vs. pattern completion.

## Introduction

The hippocampus is a key site of age-related change (e.g., Raz et al., 2005). Studies have reported not just an age-related decline in overall hippocampal volume (Raz et al., 2005; Frisoni et al., 2008), but also subfield-specific structural (Mani et al., 1986; Mueller et al., 2007) and functional (Yassa et al., 2010) alterations. In particular, studies have identified decreased cell density in the CA3 subfield (Mani et al., 1986), declining volumes of CA3/dentate gyrus (DG; Mueller and Weiner, 2009; but see Mueller et al., 2007), increased firing rates in CA3 pyramidal neurons (Wilson et al., 2005), and deficits in the maintenance of long-term potentiation in the CA3 (Dieguez and Barea-Rodriguez, 2004) in the aging hippocampus. In a recent high-resolution neuroimaging study in humans, healthy aging was associated with alterations in CA3/DG functioning (Yassa et al., 2010). Pattern completion, a process allowing the complete retrieval of a memory based on partial/incomplete information, may be critically reliant on the CA3 subfield, as outlined below. Therefore, alterations in CA3 evident in aging may result in a lessened ability to engage the auto-associative CA3 network that supports pattern completion. We suggest these age-related changes in hippocampal structure and function may coincide with corresponding decline in pattern completion abilities in healthy older adults (OAs).

Pattern completion is the ability to retrieve a memory even when only partial, incomplete, or degraded cues are available and is thought to be reliant on the CA3 region of the hippocampus (Kesner and Hopkins, 2006). O'Reilly and Norman (2002) suggest the hippocampus may support recall by orthogonalizing, or separating inputs in DG (i.e., pattern separation), and these separate representations are then stored in CA3. Via Hebbian learning, portions of CA3 form links that during memory retrieval are re-activated or reconstructed based on a partial/degraded version of the input pattern (O'Reilly and Norman, 2002). The CA3 has unique property of recurrent collaterals within itself that supports this re-activation based on partial cues. In the rodent hippocampus, dendritic synapses of CA3 pyramidal cells are largely from axon collaterals also within the CA3 (Amaral and Witter, 1989), and the CA3 receives less than one-third of inputs from extra-CA3 cells (Amaral et al., 1990). Computational models suggest that due to this recurrent connectivity of CA3 cells, the CA3 operates as a singular network, and allows for the formation of arbitrary associations from information in distinct locations in the brain. For instance, this auto-associative network might combine information about the identity of an object with its location (Rolls, 2007). This extensive intrinsic connectivity in the CA3 hippocampal subregion acts as an auto-associative network to retrieve patterns of activity, or memories, based on partial information or cues (Marr, 1971; McNaughton and Nadel, 1990; O'Reilly and McClelland, 1994; Rolls, 1996; O'Reilly and Norman, 2002; Guzowski et al., 2004; Rolls and Kesner, 2006). The recurrent collateral connections are a central element of the pattern completion process (Rolls, 2007). Rodent studies have provided converging evidence to computational models, supporting the notion that CA3 is necessary for pattern completion (Nakazawa et al., 2002; Lee et al., 2004a,b; Gold and Kesner, 2005; also see Hunsaker and Kesner, 2013 for a review).

Gold and Kesner (2005) tested CA3-lesioned rats on a delayed matching-to-place task. During the sample phase, rats removed a covering of a food well that could appear in one of five possible spatial locations. This was followed by a 30 s delay phase. During the choice phase, rats were to find the same well with the proximal block removed and thus needed to relocate it using the four distal, extramaze cues provided in the room. After they reached stable performance, rats were then given lesions to the CA3 subregion. Subsequently they were tested on the same task, with zero, one, two, three, or all four extramaze cues missing. In order to find the correct well efficiently, rats presumably have to mentally “fill in” or complete the arrangement of extramaze cues. Rats with CA3 lesions were impaired in pattern completion as indicated by a disproportionate linear increase in errors in comparison to controls as the number of available cues was reduced, suggesting the CA3 is necessary for pattern completion.

The importance of the CA3 in pattern completion is further highlighted by a study whereby Nakazawa et al. (2002) ablated the *N*-methyl-D-asparate (NMDA) receptor gene in the CA3 subregion of rodents. These rodents performed equivalent to controls on the Morris water maze task when all extramaze cues were present. However, when three out of the four extramaze cues were removed, control rodents showed the same level of recall as in the full-cue condition, whereas the mutant rodents were severely impaired on the task.

A model proposed by Wilson et al. (2006) suggests that each subregion of the hippocampus, including the CA3, undergoes age-related changes. For instance, reduced cholinergic input in aging may decrease inhibition in the CA3 auto-associative network (Hasselmo et al., 1995; Wilson et al., 2006). This disinhibition of CA3 associated with aging might result in hyperactivity of the CA3 (Wilson et al., 2006), which in turn may impair pattern completion in older adults.

The current study aimed to assess pattern completion in healthy younger and older adults using a previously validated three-dimensional virtual reality navigation task (Paleja et al., 2011) programmed using CG-Arena software (http://web.arizona.edu/~arg/data.html; Jacobs et al., 1997). We modeled our spatial pattern completion task based on a CA3-dependent rodent matching-to-sample task (used by Gold and Kesner, 2005) that manipulated extra-maze cues to systematically assess pattern completion (original paradigm from Nakazawa et al., 2002).

## Materials and methods

All study procedures were approved by the Research Ethics Board at Ryerson University. Participants included 25 younger adults (YAs; 22 female; *M*_age_ = 19.6, *SD*_age_ = 3.16, range = 17–25 years) and 25 OAs (OAs; 17 female; *M*_age_ = 70.1, *SD*_age_ = 5.29, range = 61–79 years). The YAs were undergraduate students at Ryerson University who received partial course credit for their participation in the study. The OAs were community-dwelling individuals who had participated in previous research studies in the Department of Psychology, and who received $10/hour as compensation for their time and travel. A detailed health questionnaire was administered to exclude health issues or other factors that may affect cognition or otherwise impair performance on the task. Exclusion criteria included lack of fluency in English, impaired vision, a self-reported psychological disorder, taking medication that could affect cognitive performance, as well as a history of stroke or other neurological conditions. OAs on average had significantly more years of education (*M* = 16.42, *SD* = 2.44) than younger adults (*M* = 12.72, *SD* = 1.31), *t*_(48)_ = 6.54, *p* < 0.001.

Prior to being tested on the computerized spatial navigation tasks, participants completed the Mini-Mental Status Examination (MMSE; Folstein et al., 1975) to assess general cognitive status. The average MMSE score for OAs was 28.96 (*SD* = 1.68) and scores ranged from 26 to 30, suggesting these OAs are within a normal range (Folstein et al., 1975).

The experimental procedure for the associative memory baseline and SPC tasks followed a protocol used in a previous study (Paleja et al., 2011). All tasks were performed in a circular computer-generated (CG) arena enclosed in a square room with gray walls (http://web.arizona.edu/~arg/data.html; Jacobs et al., 1997). Distances in the virtual environment were scaled such the length of one virtual stride, resembled approximately one meter and represented about 10 area units. The arena floor was gray with a diameter of 92 units and was enclosed by a circular brick wall 8 units high. The square room containing the arena was 500 × 500 area units, and had purple-colored walls 100 units high. The four external walls were each identified by a unique 100 × 60 unit fractal pattern. All fractal patterns were unique, although they had similar coloring. The center of each fractal pattern was 250 units from the corner of each adjacent wall and 65 units from the floor.

All participants completed the associative memory paradigm first and then the spatial pattern completion task. Given that there may be perceptual or working memory differences between OAs and YAs that may influence performance in the pattern completion task, the associative memory task was used as a baseline task identical to SPC but without a cue missing manipulation. This was used as a covariate in the analysis to account for differences in performance not directly related to pattern completion (see Results). The other function of the baseline associative memory task was as a training task to familiarize participants with the virtual environment and with navigation through the arena. Fourteen trials of sample-choice pairs were administered. The SPC task was administered last to ensure that the participants were first well-familiarized with the virtual environment, as well-established knowledge of the environment is particularly important for pattern completion (Kesner and Hopkins, 2006). Participants were randomly assigned to receive one of three different trial orders for each task to minimize the likelihood that the results were due to any particular sequence of target (and foil) locations (as described below).

**Figure 1:**
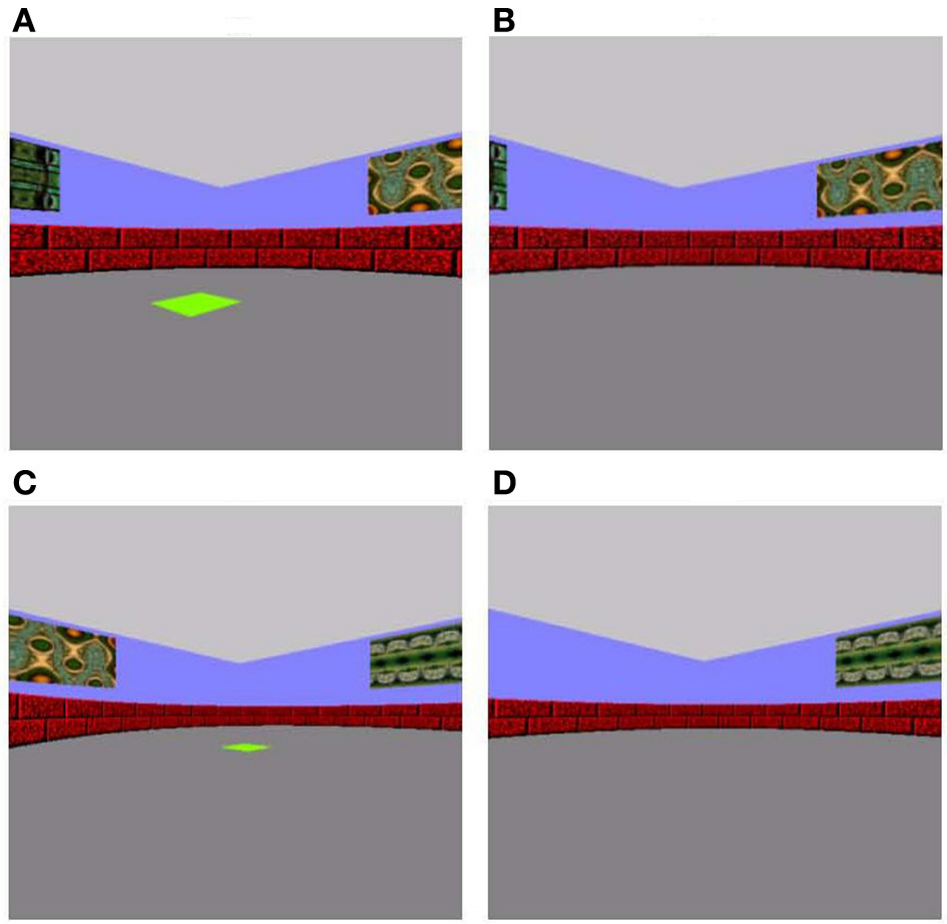
**Associative memory training task (A and B) and spatial pattern completion (C and D) tasks. (A)** In the sample phase of the training task, participants navigated using the arrow keys on keyboard to a green square. **(B)** During the choice phase, they were required to navigate to the same location the green square was previously presented in, this time in the absence of a visible green square. **(C)** The sample and **(D)** choice phases for spatial pattern completion were similar to the training phase, except the number of wall cues available was manipulated such that 0, 2, or all 4 wall cues were missing in the choice phase of spatial pattern completion.

In the sample phase of each trial of the associative memory training phase, a 5-by-5 unit bright green square was presented in one of 20 possible pre-determined spatial locations on the arena floor with varying distances from the participant's start location (Figure 1A). Participants were instructed to use keyboard arrow keys to navigate to and situate themselves on the green square. Upon reaching the square, they were to press the space bar to take them into a 5-s delay period in an empty arena. During the subsequent choice phase, participants were placed in the center of the arena in a random start orientation and required to navigate to the spatial location that had been marked in the sample phase, only this time in the absence of a visible target (Figure 1B). We placed the participant in a random orientation at every choice phase to mitigate reliance on purely perceptual processing, and maximally engage allocentric spatial processing known to critically involve the hippocampus (O'Keefe and Nadel, 1978). The participants were to use extramaze cues, including four different fractal patterns on the walls, as a guide. Participants were permitted up to 45 s to relocate the target location during the choice phase (ensuring the participant had sufficient time to navigate to the target location if they knew where it was), after which the trial would terminate. A black screen with the words “Starting next trial … ” would appear and would immediately take participants to the next study phase. There was approximately 1 s in between each trial. Twenty-one trials of sample-choice pairs were administered.

The spatial pattern completion task was modeled after Gold and Kesner's (2005) delayed matching-to-sample task. Participants began the sample trial from a designated starting position in the center of the arena facing North and traveled to a bright green square placed on the floor of the arena (Figure 1C). As with the associative training phase, targets were in one of 20 possible pre-determined locations in the arena. Subsequent to a five-second delay, participants were placed facing a random orientation to engage hippocampally dependent allocentric processing rather than reliance on perceptual cues (O'Keefe and Nadel, 1978). They were instructed to relocate the target location in the absence of a green square (Figure 1D). They were allowed to explore the maze until they reached the correct location for up to a maximum of 45 s, after which the trial would terminate, there would be a black screen with the words, “Starting next trial … ” and the task would proceed to the next study phase. The sole difference between this task and the initial associative training task was that the number of extramaze cues (fractal patterns on the walls of the arena) available was manipulated, such that 0, 2, or all 4 fractal cues were missing, whereas in the associative training task all cues were available. Across 21 study-test trials, these three possible cue conditions were repeated seven times each in a pseudorandom presentation sequence. There was approximately 1 s in between each trial. Correct answers were coded as “1” when the target was correctly located and “0” when it was not located and these values were averaged to produce a “proportion correct” score.

## Results

**Figure 2:**
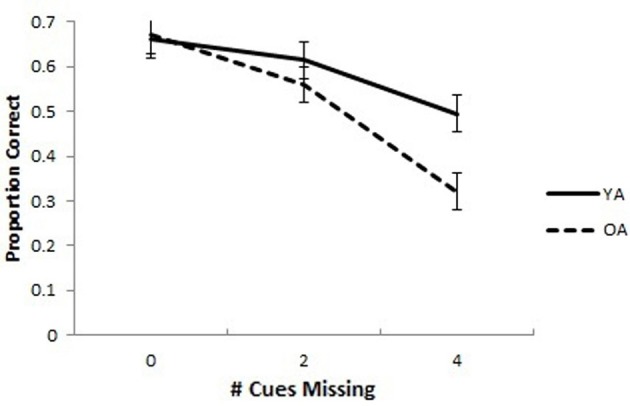
**Spatial pattern completion in younger and older adults controlling for baseline associative memory performance.** Older adults performed worse overall compared to young adults on a pattern completion task. Older adults performed similarly to young adults when 0 extra-maze cues were missing, but worse when 2 or 4 cues were missing. Error bars show the standard error of the mean.

The mean proportion of correct responses for YAs and OAs in the SPC task is presented in Figure 2. A 2 (Group: OA and YA) × 3 (Cues Missing: 0, 2, or 4) mixed model analysis ANCOVA with the proportion correct in the associative memory baseline as a covariate was conducted on the proportion of correct responses. The covariate was included to control for differences in working memory, computer experience, as well as perceptual and motor abilities that might account for group differences in the SPC task. The covariate was significantly related to SPC proportion correct, *F*_(1, 47)_ = 20.95, *p* < 0.001, partial η^2^ = 0.308. The ANCOVA yielded an interaction of Group × Cues Missing, *F*_(2, 94)_ = 3.28, *p* = 0.042, partial η^2^ = 0.065. The main effects of Group, *F*_(1, 47)_ = 3.15, *p* = 0.083, partial η^2^ = 0.063, and Cues Missing, *F*_(2, 94)_ = 0.794, *p* = 0.455, partial η^2^ = 0.017, were non-significant. Pairwise comparisons with an LSD multiple comparisons correction revealed no differences between OAs and YAs when all cues (*p* = 0.900) or 2 cues (*p* = 0.354) were missing but significant differences when all 4 cues were missing, (*p* = 0.004).

We performed identical analyses using latency as our dependent measure. These findings differed slightly from our proportion correct results above. A 2 (Group: OA and YA) × 3 (Cues Missing: 0, 2, 4) mixed-model ANCOVA with associative memory baseline latency as a covariate was conducted. As in the proportion correct analysis, the covariate was significantly related to SPC latency, *F*_(1, 47)_ = 22.71, *p* < 0.001, partial η^2^ = 0.326. The interaction between Group and Cues Missing failed to reach significance, *F*_(2, 94)_ = 2.480, *p* = 0.089. There was a main effect of Cues Missing, *F*_(2, 94)_ = 15.21, *p* < 0.001, and no main effect of Group, *F*_(2, 47)_ = 3.361, *p* = 0.073. Pairwise comparisons of the Cues Missing main effect revealed significant differences between 0 cues missing (*M* = 23.93, *SD* = 8.34) and 2 cues missing (*M* = 26.82, *SD* = 8.61), *p* = 0.032, and 2 cues missing and 4 cues missing (*M* = 34.00, *SD* = 7.17), *p* < 0.001.

## Discussion

In the present study, we aimed to assess spatial pattern completion abilities in older adults by systematically manipulating the availability of extramaze cues in a virtual environment. This navigational task was based on a rodent matching-to-sample task sensitive to CA3 function (Gold and Kesner, 2005). We found that overall, performance decreased as a function of decreasing cue availability. We hypothesized that older adults would perform worse than younger adults as cue availability decreased, due to an increased reliance on pattern completion to successfully perform the task. The data suggest spatial pattern completion abilities may decline with age. Importantly, group differences were not due to the older adults' difficulty with task instructions, differential motor or perceptual abilities, or inexperience with computer use. First, older adults performed equivalently to young adults when all cues were available. Second, baseline performance with identical task demands but without a pattern completion manipulation as a covariate on the pattern completion task produced differential performance profiles for younger and older adults, with older adults performing disproportionately worse as cue availability decreased. We did not observe exactly the same pattern of findings for latency. This may be due to the fact that the trials timed out at 45 s, and this may have obscured true differences that our proportion correct measurement captured.

There is a growing body of evidence showing pattern separation declines in aging (Toner et al., 2009; Burke et al., 2010, 2011; Stark et al., 2010; Yassa et al., 2010, 2011; Holden et al., 2012; see Holden and Gilbert, 2012 for a recent review), and authors have suggested there may be a corresponding bias toward pattern completion (Toner et al., 2009; Stark et al., 2010; Yassa et al., 2010, 2011). Hunsaker and Kesner (2013) highlight a common misconception in the literature, according to which pattern separation and pattern completion are two ends of a single continuum. In fact, the two processes rely on different neuroanatomical structures (pattern separation relies on DG, and pattern completion on CA3) and occur at temporally distinct processing stages (pattern separation at encoding, and pattern completion at retrieval; Hunsaker and Kesner, 2013). Therefore, an impairment in one does not necessitate an improvement in the other (see Hunsaker and Kesner, 2013 for a review). If a bias away from pattern separation and toward pattern completion does exist in older adults, it does not necessarily manifest itself in intact behavioral pattern completion performance, as evidenced by poorer performance by older adults here. Our data suggest that the auto-associative network supporting pattern completion may be impaired in older adults, consistent with studies showing healthy aging to be associated with CA3 abnormalities (Mani et al., 1986; Mueller and Weiner, 2009; Yassa et al., 2010).

A potential concern might be whether the processes tapped into with this task are indeed hippocampally dependent. The short delay between sample and choice phases (30 s) may recruit working memory processes rather than long-term episodic memory, the former which has been shown by studies with temporal lobe amnesia patients to be less hippocampally-dependent (e.g., Baddeley and Warrington, 1970). However, there is recent evidence to suggest short-term memory involves the hippocampus (Kesner and Hopkins, 2001; Kessels et al., 2001; Piekema et al., 2006). For instance, one study found spatial working memory deficits in hippocampally lesioned patients (Kessels et al., 2001). In another study, hypoxic patients with bilateral hippocampal pathology were tested on short-term memory for an item, spatial item distance, or temporal duration. Hypoxic participants were impaired in short-term memory for duration and spatial distance information but showed less impairment for visual item information (Kesner and Hopkins, 2001). Other findings suggest the right hippocampus, in particular, may be important for the maintenance of object-location associations over a short duration, consistent with its function over a longer duration (Piekema et al., 2006). Therefore, although theoretical (keeping the task analogous to those used in rats) and logistical (ensuring an adequate number of trials in each cue missing condition were collected within a session without fatiguing the elderly participants) factors influenced our choice of shorter delays, the use of a short delay in our task does not necessarily mean our task is independent of the hippocampus, and may in fact engage or be reliant on the hippocampus. Further, our use of the associative memory baseline as a covariate for our pattern completion task controlled for inherent group differences in working memory, perception, motor abilities, and computer experience since these are equally required in both tasks.

An important question that should be addressed in future research is to what extent age-related declines in pattern separation may contribute to the age-related deficit in pattern completion that we observed. Presumably, the encoding of a fine-grained representation (i.e., pattern separation) during the sample phase becomes increasingly important as the availability of cues declines during the choice phase. In this study, we examined pattern completion by manipulating cue availability at retrieval, but it is worthy of consideration that other processes, including deficient pattern separation at encoding, may contribute to older adults' deficit in pattern completion. However, given the extensive literature showing CA3 abnormalities in aging, including CA3 hyperexcitability, we speculate pattern completion deficits are not due entirely to impaired pattern separation and rather reflect a more fundamental issue with abnormal functioning of the CA3 autoassociative network.

This study used a novel, immersive virtual reality paradigm analogous to a CA3-sensitive rodent task (Gold and Kesner, 2005) to systematically assess spatial pattern completion in older adults. We provided evidence that older adults are impaired in spatial pattern completion compared to younger adults, and that their performance declines as pattern completion demands increase. These findings, in conjunction with the existing pattern separation findings, may shed light on the nature of associative memory deficits reported in older adults (e.g., Naveh-Benjamin, 2000; Naveh-Benjamin et al., 2003; Old and Naveh-Benjamin, 2008). In particular, both pattern completion and pattern separation difficulties in older adults may be at the core of broader age-related associative memory changes.

### Conflict of interest statement

The authors declare that the research was conducted in the absence of any commercial or financial relationships that could be construed as a potential conflict of interest.
